# Adherence by Dutch Public Health Nurses to the National Guidelines for Tuberculosis Contact Investigation

**DOI:** 10.1371/journal.pone.0049649

**Published:** 2012-11-14

**Authors:** Christiaan Mulder, Janneke Harting, Niesje Jansen, Martien W. Borgdorff, Frank van Leth

**Affiliations:** 1 KNCV Tuberculosis Foundation, The Hague, The Netherlands; 2 Center for Infection and Immunity Amsterdam (CINIMA), Academic Medical Center, University of Amsterdam, Amsterdam, The Netherlands; 3 Department of Public Health, Academic Medical Center, University of Amsterdam, Amsterdam, The Netherlands; 4 Department of Infectious Diseases, Public Health Service, Amsterdam, The Netherlands; 5 Department of Clinical Epidemiology, Biostatistics and Bioinformatics, Academic Medical Center, University of Amsterdam, Amsterdam, The Netherlands; 6 Department of Global Health, Academic Medical Center, University of Amsterdam, Amsterdam Institute for Global Health and Development, Amsterdam, The Netherlands; McGill University, Canada

## Abstract

**Objectives:**

To assess whether public health nurses adhered to Dutch guidelines for tuberculosis contact investigations and to explore which factors influenced the process of identifying contacts, prioritizing contacts for testing and scaling up a contact investigation.

**Methods:**

A multiple-case study (2010–2012) compared the contact investigation guidelines as recommended with their use in practice. We interviewed twice 14 public health nurses of seven Public Health Services while they conducted a contact investigation.

**Results:**

We found more individuals to be identified as contacts than recommended, owing to a desire to gain insight into the infectiousness of the index case and prevent anxiety among potential contacts. Because some public health nurses did not believe the recommendations for prioritizing contacts fully encompassed daily practice, they preferred their own regular routine. In scaling up a contact investigation, they hardly applied the stone-in-the-pond principle. They neither regularly compared the infection prevalence in the contact investigation with the background prevalence in the community, especially not in immigrant populations. Nonadherence was related to ambiguity of the recommendations and a tendency to act from an individual health-care position rather than a population health perspective.

**Conclusions:**

The adherence to the contact investigation guidelines was limited, restraining the effectiveness, efficiency and uniformity of tuberculosis control. Adherence could be optimized by specifying guideline recommendations, actively involving the TB workforce, and training public health nurses.

## Introduction

Investigating the contacts of patients with pulmonary tuberculosis (TB), or index cases, is considered one of the cornerstones for TB control in low-incidence countries [1], [2], [3]. The objectives of a contact investigation (CI) are to identify and treat patients with secondary TB to reduce further transmission and to identify and treat infected contacts to prevent progression to active TB and further transmission. In the Netherlands, the intent of the 2007 national guidelines for CI is to guarantee an effective, efficient and uniform process across Public Health Services (PHSs). Within these organizations, public health nurses (PHNs) have the primary responsibility for conducting the CIs under the supervision of a physician specialist. In turn, the PHSs routinely report on the outcomes of CIs in the Netherlands Tuberculosis Register; these data are evaluated at the national level and help direct policy development.

Three important steps in a CI are: identifying contacts, prioritizing contacts for testing, and scaling up an investigation (Table 1). According to the national guidelines, individuals should be identified as contacts if they have been exposed to an index case and have a substantial risk of being infected. This risk depends on the infectiousness of the index case, the period of exposure (frequency and duration) and the location of exposure (e.g., small, dark and warm areas). Once identified, individuals should be prioritized for testing based on their risk of infection as close, casual or community contacts (Table 2). Vulnerable contacts, like children and immunocompromised individuals, should always take priority. According to the stone-in-the-pond principle, testing should be started in close and vulnerable contacts. If the number of close contacts is small, casual contacts may be tested simultaneously. Testing should be scaled up to casual and, subsequently, community contacts, only if the prevalence of infection in the tested contacts is markedly higher than the expected background prevalence in the community (Table 3). Additional criteria are the expected prevalence in these less prioritized contacts and a consideration of the costs and benefits [4], [5].

**Table 1:** The three steps during a CI according to Dutch national guidelines: identifying contacts, prioritizing contacts, and scaling up a CI.

Description	Criteria	Level of evidence
*Identifying contacts*	*Infectiousness*	
Individuals who have been exposedto the index case and have a substantialrisk of being infected are eligible to beidentified as a contact. This risk is based on the infectiousness of the index case,the period of exposure and location of exposure.*	Clinical characteristics of the index case: having sputum smear positive TB, cavernous TB, poor coughing behavior.	Risk of infection is significantly associated with smear positive TB and cavitary lesions [21], [22], [23], [24], [25]. Infectiousness is positively correlated to poor coughing behavior [26].
	*Period of exposure*	
	Prolonged and frequent exposure to the index case during the period of infectiousness. The standard period of infectiousnessis set at three months prior to the diagnosis of TB.	Increased hours of exposure to the index case is associated with being infected [21] The standard period of three months is based on expert opinion [27].
	*Location of exposure*	
	Exposure to the index case in a location which is small, dark,warm, humid, crowded, and poorly ventilated.	The volume of air shared between the index case and contacts dilutes the infectious droplets, but this has not been validated entirely [27].
*Prioritizing contacts*		
Contacts should be prioritized for testing by classifying them as close, casual or community contacts.	Contacts should be prioritized in conformity with theclassification table which is based on a combination of the period andlocation of exposure (see Table 2).	The classification table is based on the guidelines concerning contact investigation published by CDC in 2005 [27]. These CDC guidelines are evidence-based, but also rely on expert opinion.
	Close contacts and vulnerable contacts, like children and immunocompromised contacts, have priority for testing.	Evidence-based. Immunocompromised contacts and vulnerable contacts have an increased risk of progression to (severe) active TB [27].
*Scaling-up a CI*		
Stone-in-the-pond principle		
	Testing should be started in the close and vulnerable contacts.When the number of close contacts is too small* to make reliableestimates regarding the prevalence of infection, the casual contactsmay be tested simultaneously with the close contact andvulnerable contacts.	Not evidence-based.
	Testing should be scaled up to the casual, and subsequently community, contacts when the prevalence of infection in thetested contacts is markedly higher* than the backgroundprevalence in the community (Table 3) and a higher* prevalencewill be expected in these less prioritized contacts as well.	The prevalence of infection in contacts has shown to diminish when the level of exposure to the index case became less [5].
	The background prevalence is age-specific and in TheNetherlands is reported only for the native population. It ranges fromless than 1% for people born after 1965 to >5% for people bornbefore 1945 (Table 3).	This background prevalence was assessed among army recruits when they were aged 20 years [4].

Abbreviations: CDC: the Centers for Disease Control and Prevention, CI: contact investigation, TB: tuberculosis.

* Not further specified in guidelines.

**Table 2:** Prioritizing contacts for testing according to the classification table as suggested by the national guidelines.

			Period of contact			
Intensity	Size of location ofexposure is comparablewith*	Estimated volume or location	Prolonged	Less prolonged		
			Daily or>48 hours	Weekly or 6–48 hours	Incidentally or 1–6 hours	Sporadic or <1 hours
Close	Car	<5 m^3^	1	1 and 2	2	2
	Room	10–30 m^3^	1	2	2	2 or 3
Less close	Classroom/office**	100–200 m^3^	2	2 or 3	3	3
	Closed room bigger than ahouse**	>200 m^3^	2	3	3	3 or other

(1 = close contact, 2 = casual contact, 3 = community contact).

* Accounting for survival of *Mycobacterium tuberculosis* (air refreshment and circulation) and ventilation.

** Use ‘room’ if the contact have been exposed to the index case within a distance of <1–2 m in this location.

**Table 3:** Estimated age-specific background prevalence of LTBI in native population in 2005 as reported in the national guidelines.

Year of birth contact	% tuberculin skin test positive†
1945–1954	5%
1955–1964	2%
1965–current	1%
Overall	2,40%

† Not vaccinated with bacille Calmette-Guérin.

The role of guidelines in standardizing clinical practice has been debated [6], [7]. They may serve as a coordinating tool, but do not resemble a blueprint. For instance, decision criteria are not always explicitly stated in the guidelines [6]. Although largely evidence-based, various recommendations of the Dutch guideline for CI are formulated rather loose and lack adequate specification (Table 1), leaving room for differential interpretations. Another criticism is that evidence used in guidelines often lacks contextual information regarding social, cultural and political dimensions which are interrelated to the daily practice [7]. Although several recommendations of the Dutch guideline for CI rely on consensus between experts as well (Table 1), it may be expected that they interact with previous perceptions and experiences of healthcare workers [7]. Only when guidelines are actually being used, it may become clear what criteria are not explicitly stated and how recommendations interact with pre-existing practices [6]. So, to have the national guidelines for CI implemented in daily practice, it is important to explore how they are actually applied, or adhered to, by the professionals they are meant for.

Research regarding adherence to CI guidelines has been limited. In Australia, a review of individual records on CI concluded that the diagnostic guidelines were outdated and that adherence to these guidelines was poor [8]. A similar conclusion was reached in the United States by a review of aggregate program management reports, while also reporting challenges with respect to the identification of contacts [9]. Two qualitative studies, both from the United States, reported wide variety in CI protocols and definitions [10], [11]. These studies revealed barriers in identifying and prioritizing contacts and in scaling up a CI. Among these were the absence of straightforward definitions, insufficient training of field workers, misunderstandings about the importance of identifying contacts and patient-level barriers, such as mistrust by patients (8–9). None of these studies, however, reported how decisions were made regarding the three essential steps at the level of an individual CI.

The objective of our study was to assess whether PHNs adhered to the national guidelines at the individual CI level and to explore which factors influenced the process of identifying contacts, prioritizing contacts for testing and scaling up a CI.

## Methods

### Ethics Statement

We were exempted from ethical approval from the Netherlands Central Committee on Research Involving Human Subjects, because no direct contact with patients with TB was required.

### Design and Participants

For our multiple-case study (n = 14), we purposely selected seven PHSs (out of a total of 28), because of their locations in both urban and rural areas that covered five out of eight regions responsible for TB control in the Netherlands. A total of 25 PHNs from all seven sites were invited to participate; 14 became participants, constituting one fifth of all Dutch PHNs working in TB control, and their written or oral (via telephone) informed consent was obtained. Nonparticipants either had not recently initiated a CI or had no interest in the study. This may have induced participation bias. We think, however, that our sample was a fair representation of PHNs in Dutch TB control, as the entire group is relatively homogeneous with respect to sex, age, ethnicity and working experience.

### Data Collection

Data were collected between September 2010 and February 2012. One investigator (CM) interviewed each PHN twice concerning a single CI. The total number of evaluated CIs was 14 and each CI was around a different TB patient (Table 4). The first interview was conducted either via telephone or face-to-face directly after a CI was initiated. The second interview was conducted face-to-face when it was concluded. For each of the two semi-structured 1-hour interviews, we used a topic list reflecting the content of the CI guidelines (Table 5). The topic list enabled us to use probing questions and discuss the process of a CI meticulously. The topic list was reviewed by experts working in Dutch TB control, and pilot tested at a PHS that did not participate in the study. For the first interview we used the first part of the list which addressed the identification of contacts and their subsequent prioritization for testing. For the second interview we used the second part which addressed the application of the stone-in-the-pond principle and issues related to scaling up a CI. As the focus of our study was on identifying contacts, prioritizing contacts for testing and scaling up a CI, we considered information about diagnostics used in the CI as outside the scope of the present paper.

**Table 4:** Association of scaling up a CI with the number of close, casual and community contacts evaluated and detected with LTBI found in the 14 CIs.

			Close contacts			Casual contacts			Community contacts		
Case	PHN/PHS	Ethnicity indexcase	Number of contacts tested	Number of contacts with LTBI	Scaled up?	Number of contacts tested	Number of contacts with LTBI	Scaled up?	Number of contacts tested	Number ofcontacts with LTBI	Scaled up?
1	1/2	Native	3	1	Yes^f^	1†	0	Yes^i^	1	0	No
2	2/2	Immigrant	1	0	Yes^b^	38†	0	Yes^i^	3†	0	No
3	3/2	Immigrant	32	0	Yes^d^	8†	0	No	–	–	–
4	4/2	Immigrant	22	4	Yes^e^	11†	0	No	–	–	–
5	1/3	Immigrant	6	0	Yes^d^	4	0	Yes^i^	1†	0	No
6	2/3	Native	23	6	Yes^a^	63	2	Yes^h^	1660	23	Yes
7	1/4	Immigrant	23	2	Yes^a^	1	0	No	–	–	–
8	1/5	Immigrant	5	1 (+1 TB)	Yes^b^	28†	0	No	–	–	–
9	2/5	Immigrant	25	5	Yes^e^	5†	0	No	–	–	–
10	1/6	Immigrant	5	1 (+2 TB)	Yes^a^	129	8 (+1 TB)	No^k^	–	–	–
11	2/6	Immigrant	17	0	No^c^	–	–	–	–	–	–
12	3/6	Immigrant	21	5 (+2 TB)	Yes^e^	114†	8	No^j^	–	–	–
13	1/7	Immigrant	26	5	Yes^a^	4	1	Yes^h^	0	–	No
14	1/8	Native	1	0	Yes^g^	7†	0	Yes^i^	23	0	No

† Tested concurrently with the previous group of contacts.

a Scaled up according to guidelines because the decision was based on the prevalence of infection among the close contacts.

b Scaled up according to guidelines because the PHN considered the number of close contacts too small to accurately assess the prevalence of infection.

c Correctly not scaled up because decision was based on prevalence of infection among the close contacts.

d Incorrectly scaled up to casual contacts since no infection was found among the close contacts.

e Incorrectly scaled up since casual contacts were tested concurrently with close contacts.

f Incorrectly scaled up since casual contact was considered a ‘test case’.

g Incorrectly scaled up since casual contacts were anxious.

h Scaled up to community contacts according to guidelines because the decision was based on the prevalence of infection among the casual contacts.

i Incorrectly scaled up to community contacts since no infection was found among the casual contacts.

j Incorrectly not scaled up to community contacts although prevalence of infection among casual contacts was high.

k Not scaled up to community contacts because according to PHN there was no well defined group of community contacts.

**Table 5:** Questions of the topic list used to explore how public health nurses identified and prioritized contacts and scaled up a CI.

	Questions	Probing questions
Identification of contacts	How did you identify the contacts?	Did someone else than the index case assisted with naming the contacts?
	Did you measure the level of exposurebetween the contacts and the index case?	How? How did you measure the frequency/duration/intensity of exposure?
	To what extend is the list of contacts completedo you think?	Why do you think it is/is not complete? What efforts did you undertake to identify all contacts?
	How is the relationship between you and theindex case?	How was the willingness of the index case to name his/her contacts? Did you experience any barriers in identifying contacts?
Prioritization for testing	How were the contacts prioritized in thiscontact investigation?	When were the contacts prioritized? Who decided on this prioritization? Did you discuss this with the physician specialist? Why/why not?
	To what extent did you experience difficultieswith prioritizing contacts?	What were the reasons for experiencing these difficulties?
	How did you differentiate between close andcasual contacts?	Which criteria you considered most important? Duration of exposure? Frequency? Intimacy? Other criteria? Why?
	How did you differentiate between casual and community contacts	Which criteria you considered most important? Duration of exposure? Frequency? Intimacy? Other criteria? Why?
Scaling up	Were casual contacts evaluated?	How and why was decided to test casual contacts? Who made this decision
	Were casual contacts tested concurrently or subsequently of close contacts?	For what reason?
	Were community contacts tested?	How and why was decided that community contacts were tested? Who decided this? If not: How and why was decided not to test community contacts? In what hypothetically situation would you have scaled up to test casual/community contacts? Which contacts would then have been tested?

PHNs were encouraged to describe and to explain their current practice. To discourage socially desirable answers, it was explicitly stated that the study was exploratory and was not intended as a clinical audit. The anonymity of the PHNs was guaranteed. By prospectively following the CIs, we could have induced a Hawthorne effect, meaning that subjects’ behavior is altered by awareness of being observed [12]. As most PHNs had more than 10 years of experience, we believe that the risk for this bias was limited. We used semi-structured interviews rather than structured observations, because we expected CIs to last for an extensive period (half a year). It would then have been difficult to capture all relevant data since decisions were made during the course of a CI, given that we studied 7 PHSs throughout the country. We are confident we reached data saturation since the last interviews in our study did not reveal new information.

### Analysis

Each interview was transcribed verbatim, and a summary was returned to the PHN to check for inconsistencies (member checking). Transcripts were imported in NVivo 8 (QSR International, Victoria, Australia) to support systematic within-case and cross-case content analysis [13]. We used a semi-structured coding list that reflected the recommendations in the CI guidelines (pre-structured coding), as well as the reasons for adhering or not adhering to these recommendations (open coding). Two researchers (CM and JH) independently coded the first interview to be analyzed; after this analysis the coding structure was further specified and completed. They then coded two more interviews, observing no major coding discrepancies. All other interviews were coded by CM. The final cross-case analysis allowed identification of common themes of adherence or nonadherence across the CIs.

Of the authors and participating PHNs, only one researcher (NJ) was involved in the writing of the guidelines. She was, however, not involved in any coding of the transcripts. None of the authors were actively involved in conducting CIs.

## Results

### Study Participants

The PHNs conducted on average 2 to 25 CIs each year. Eight of the 14 PHNs had worked in TB control for 10 years or more (range 2–26 years).

### Index Cases

Nine of the 14 index cases involved males, 11 were 35 years or younger, and 11 were immigrant (either first- or second-generation). All had culture confirmed pulmonary TB, and 11 of 14 had acid fast bacilli in their sputum (smear-positive).

### Identifying Contacts

Most contacts were identified in accordance with the guidelines; however, identification was challenging, because index cases were not always able to recall or willingly to share contact information. *“Apparently, the index case had a boyfriend. She purposely didn’t tell me that.”* [PHN 3, PHS 2] [All direct quotations from PHNs are English translations.].

The PHNs tended to indentify more individuals as contacts than recommended for three reasons. First, most PHNs tried to make a comprehensive assessment of the level of the infectiousness of the index case. In their view, this meant including as many individuals as possible as a contact, instead of including just those at substantial risk for infection. *“Ultimately, the more contacts you test, the more insight you get into the infectiousness.”* [PHN 1, PHS 8]. For that reason, in several CIs, the period of potential infectiousness of the index case was prolonged. This enabled the PHNs to include additional individuals as contacts, although evidence of transmission was lacking: *“You are never sure for how long and when the patient was contagious.”* [PHN 2, PHS 3] In CIs involving immigrant contacts, native Dutch contacts with limited exposure were included since assessing the prevalence of infection among immigrants was often considered unreliable because of the high a priori risk of infection in their country of origin. Infection among the native Dutch contacts was used as a proxy to interpret the infectiousness of the index case. “*There were many foreign-born contacts in this contact investigation, so therefore we could not have determined accurately his [index case] infectiousness, because we could not determine whether the index case was the source of the infections. Therefore we decided to include his colleagues [casual contacts], including some native Dutch, straightaway.”* [PHN 2, PHS 5].

Second, the PHNs hesitated to miss secondary cases or infected contacts because of the potential consequences of TB for individual contacts and of further transmission: *“I do not distinguish too rigorously which individuals are eligible as contacts and which individuals are not, because I’m dealing with a school setting. In such a setting I do not want contacts that might progress to disease. So, I include many contacts generally.”* [PHN 4, PHS 2] The PHNs also feared consequences for their job performance and/or the professionalism of the PHS: *“If infections were found in the near future that could be linked to a CI which had not been conducted carefully, then, well, I imagine that this would lead to a formal complaint.”* [PHN 4, PHS 2].

Third, the PHNs tried to avoid problems with potential contacts. For instance, individuals who came to the PHS because of anxiety about possible exposure were often identified as contacts, although the risk of transmission was considered low. *“What was known from the CI so far [prevalence of infection was perceived low] was not a reason to identify and test these two [community] contacts.”* [PHN 1, PHS 2] Individuals who were expected to cause conflicts were similarly more likely to be included in the CI: *“Sometimes someone extra gets in, because, if he didn't get in, he would raise hell about it at the school.”* [PHN 2, PHS 3].

### Prioritizing Contacts

Prioritizing contacts for testing was interrelated with the step of identifying the contacts. Contacts who were included in the CI for other reasons than recommended by the guidelines had priority to become tested. Half of the PHNs said they used the classification table to prioritize contacts (Table 2). They acknowledged its usefulness, but they had difficulty capturing exposure information. The table was perceived as less useful because the actual exposure locations, e.g. ‘house’, were not always indicated in the table and locations in the table, e.g. ‘car’, were rarely mentioned in the CIs. Not using the table was related to experiencing practical difficulties during the CI. For example, prioritizing contacts from a specific contact group, like family members or colleagues, in different categories was often deemed unpractical, and could confuse contacts, because it would imply that they were tested differently. “*I had the sense that it could result in concerns on the workplace if I had decided to define some of her colleagues as close and the rest as casual contacts. So instead I considered them all as casual contacts and invited them all.” [PHN 2, PHS 2].*

The PHNs who did not use the table classified contacts based on their routine approach, reflected by nonspecific terms like ‘daily’, ‘frequent’, or ‘intensive’. They also used criteria other than the level of exposure as proposed by the classification table, e.g., the severity of the symptoms of the index case. These additional criteria were perceived as important parameters of the infectiousness of the index case. Lack of knowledge of national definitions for differentiating types of contacts also led to classification difficulties: *“The less close the contacts, the less I can recall which criteria I should use to differentiate between casual and community contacts.”* [PHN 3, PHS 6]. In particular the PHNs with much work experience (≥15 years) did not use the classification table. These PHNs also did not discuss the classification of the contacts with the physician specialist of the PHS.

In some CIs, the PHNs deliberately gave certain contacts a higher priority than indicated by the table. They were guided, first, by the desire to gain insight into the infectiousness of the disease, and, second, by concerns about the well-being of the individuals. For example, one PHN considered it more efficient to have classmates screened immediately: *“What is the benefit of classifying these contacts as casual contacts? Then you have to wait for two months before you can test them because of the incubation period.”* [PHN 3, PHS 2] Third, certain contacts were considered to be “key contacts” who were assigned greater weight than “ordinary contacts”. For example, a general practitioner was considered a casual contact based on exposure data. Due to his profession however, he was tested concurrently with the close contacts. His positive test results were decisive in inviting all his patients to be tested. Fourth, higher priority was based on the mistaken belief that in each specific contact group, such as family or colleagues, a few individuals should be considered close contacts, whereas based on exposure data they were not: *“According to the guidelines, his closest colleagues in the same department were actually casual contacts, but in the group of work contacts, I classified them as close contacts.”* [PHN 1, PHS 5] Finally, anxious contacts were often given testing priority, so the PHSs could avoid potential conflicts: “*This type of diseases always results in a certain degree of panic in people. They can become quite compelling” [PHN 1, PHS 2].*

In conformity with the recommendations, vulnerable individuals, like children, were given priority in the CIs. Immunocompromised individuals, who also should have been given priority, could not be identified due to the procedures for assessing immune status. The status was not assessed for all contacts prior to testing, since this was regarded as inefficient: *“It would have taken us days to know the immune status of all these contacts prior to testing them. Instead, we asked them about their immune status while testing them.”* [PHN 2, PHS 3] This means that immunocompromised individuals may have been incorrectly excluded from testing.

### Scaling Up Contact Investigation

On the basis of the guidelines, six CIs were correctly scaled up to casual contacts (Table 4). In two of these CIs, the number of close contacts was considered too small to accurately assess the prevalence of infection; in the other four, the stone-in-the-pond principle was adequately applied. In another case the PHN correctly did not scale up the CI to casual contacts because no infections were found among the close contacts. The other seven CIs were incorrectly scaled up to casual contacts. Two CIs were scaled up to community contacts in conformity with the guidelines. In four CIs, scaling up to community contacts was not in line with the recommendations.

Inappropriate decisions with respect to scaling up a CI were first of all interrelated with the flaws during the steps of identifying and prioritizing contacts, such as gaining additional insight into the infectiousness of the index case, prevent anxiety among potential contacts and prevailing the consequences of missing secondary cases or infected contacts for the individual, the PHNs themselves, and the PHS. As a consequence, the PHNs had the tendency to scale up a CI so more contacts could be evaluated.

Three other reasons for inappropriate decisions emerged. First, the guideline recommendation that scaling up should be considered if the observed prevalence of infection was markedly higher than the background prevalence in the community appeared to be ambiguous. That is, how much higher is left open to interpretation. Second, the available criteria for scaling up were not always applied. That is, some PHNs did not use the prevalence of infection at all. Those PHNs based their decision to scale up on their personal interpretation of the results, e.g. by interpreting absolute numbers of infected contacts. Also, PHNs did not always compare the prevalence of infection with the expected background prevalence in the community, for instance, because the PHN did not use the prevalence table (Table 3). *“I know it is possible to calculate the background prevalence […] to compare it with the prevalence in the tested contacts, but I do not know these numbers by heart. So, in daily practice I […] rather use my routine.”* [PHN 1, PHS 7] Consequently, one PHN decided not to scale up a CI to community contacts, although the prevalence in the casual contacts was five times higher than the background prevalence. Additionally, this PHN underestimated the expected prevalence of infection in the community contacts. If interpreted correctly, both criteria for scaling up were met and should have been reason to scale up. Third, the CI guidelines provide the background prevalence of infection for only the native population. In CIs that included immigrant contacts, PHNs collected difficult-to-interpret anamnestic data, like travel data, to decide whether an infection was attributable to recent transmission. They also used findings among native contacts as a proxy for the infectiousness of the index case. This difficulty may have caused the PHNs to miss at-risk contacts.

### Individual Versus Population Health

The considerations of the PHNs concerning adherence to the guidelines reflected one common dilemma: whether they were primarily responsible for the health of the individual or that of the population: *“There is always a bit of tension, I mean, you matter to the patient, but at the same time, you are important to the population. […] those two things can come into conflict with each other.”* [PHN 4, PHS 3] Such conflicts are illustrated by decisions in which population health considerations gave way to possible consequences for the individual, resulting, for instance, in identifying more individuals as contacts and in deliberately giving certain contacts a higher testing priority. These decisions were sometimes at odds with the explicit opinion of the PHN: *“But in fact, we [PHNs] are not responsible for the patient. […] The PHS as an organization [is responsible] for the population health.”* [PHN 3, PHS 2] However, the individual health perspective, perhaps unconsciously, prevailed: *“Therefore my job is to identify and protect the contacts, especially the close contacts.”* [PHN 3, PHS 2].

## Discussion

Our study on CIs in TB control revealed that Dutch PHNs do not always adhere to the recommendations of the national guidelines. Most PHNs tended to identify more individuals as contacts than recommended to gain additional insight into the infectiousness of the index case and to prevent anxiety among potential contacts. Apprehension regarding the consequences for the individual, the PHNs themselves, and the PHS of missing secondary cases or infected contacts also played a role. In prioritizing contacts for testing, the PHNs found that the criteria of the classification did not fully cover circumstances in daily practice, and they preferred to use their own routine approach. In addition, certain contacts were deliberately given a higher priority than recommended, which was legitimized by concerns about the individuals’ well-being. Similar reasons underlay incorrect decisions about scaling up a CI. Correct application of the stone-in-the-pond principle was hampered by difficulty using the infection prevalence in the CI in relation to the background prevalence in the community, and even more so by absence of the background prevalence for immigrant populations. Dilemma’s in following the guidelines were related to the ambiguousness of some of the recommendations, but also to the tendency to act from an individual health care position rather than a population health perspective.

Our findings agree with those of previous studies on the adherence to clinical guidelines generally [14] and CI guidelines particularly [8], [9], [10], [11]. Guideline adherence was suboptimal [8], [9], and nonadherence could be explained by undefined or poorly defined recommendations, divergent opinions, such as how to prioritize contacts and how to scale up a CI, and inadequate competencies of field workers, and index case-related or contact-related obstacles [10], [11]. Our study clearly showed how ambiguous criteria leave room for differential interpretations [6], and how these interpretations are fostered by reasons derived from individual perceptions and previous experiences of health care workers [7]. That is, the guidelines recommend what the process of a CI should consist of, but lack information on how to manage this process. In our study, the PHNs faced quite some dilemmas while applying the guidelines for CI in practice. Several of those dilemmas clearly reflected the absence of contextual information [7]. For instance, in prioritizing contacts for testing, the PHNs assumed that the proper application of recommendations would create confusion, increase anxiety and generate hostility amongst potential contacts. Similarly, in scaling up a CI, the PHNs tended to assign greater weight to potential contacts with important societal roles, such as a general practitioner. Our findings therefore reinforce previous pleas for the inclusion of contextual information regarding social dimensions which are interrelated with daily practice [7].

In addition to previously identified barriers to adherence to guidelines for CI in TB control [10], [11], our study revealed that a tension between the perspectives of individual health and population health may be another crucial factor influencing adherence.

Although the PHNs in our study fully acknowledged their role in public health, they may not have been fully aware of the implications of their actions. For instance, by including as many contacts in the CI as possible and by deliberately giving certain individuals a higher priority for testing, the PHNs believed they contributed to population health goals, whereas they were focused on the health of the individuals involved. Given the risks of adverse effects related to overdiagnosis, this practice was not per se beneficial to the health of the tested individuals [15]. First, working from a purely population health perspective may be difficult, as individual health and population health cannot be seen as absolute and independent concepts [16]. Strengthening the population health perspective may require an increased understanding of the way in which CIs, as recommended by the guidelines, follow a classical pathway of transmission. Regular feedback from national level on registered CI outcomes could for instance help the PHNs to acquire such an understanding. Second, a population health perspective may require that PHNs, as well as potential contacts, accept that an individual risk of getting tuberculosis is similar to the background prevalence. Good education prior to testing of anxious contacts about what to do in case of complaints and about the intensity of the preventive therapy might help in diminishing the demand for testing, which again emphasizes the need for information about the social context in the CI guidelines. Third, the tension between the individual and population health perspective may be partly due to the lack of contextual information in the CI guidelines on the hierarchical organization of the PHS. The perceived negative consequences of missing infected or diseased contacts for their job performance and/or the professionalism of the PHS, may originate from the way the PHNs are supervised and reviewed by their superiors, which are often physician specialists. Because of their biomedical background, these specialists perhaps lack a strong population health perspective, for which reason CIs may be focused on the individual health of the contacts, rather than on considerations of the effectiveness and efficiency from a population health perspective.

As a primary consequence of the suboptimal adherence to the CI guidelines, the CIs conducted in the Netherlands are less uniform, effective and efficient than preferred. Thus, revising and implementing the Dutch CI guidelines in conformity with the recently formulated European consensus statement on CI in low-incidence countries [1] will require great effort, significant resources and targeted activities, such as intensive collaboration between policy makers and the TB workforce and organizing training and follow-up meetings [11]. Our study highlights that specifically the stone-in-the-pond principle warrant special attention. This principle could only remain useful in low-incidence countries if accurate data regarding the prevalence of infection in certain immigrant populations will be adopted [17], especially since immigrants are expected to be overrepresented in TB epidemiology the coming decades. [18]. Even when PHNs adhere to the guidelines regarding scaling up, the validity of the stone-in-the-pond principle could be argued on the basis of the small number of detected infections, especially since the infections might have been detected among incorrectly identified and prioritized contacts.

In the revision of the CI guidelines, the PHNs should be involved to make the guidelines workable and to account for social and organizational contextual information [7]. For instance, PHNs could be invited to share the practical dilemmas they face while acting from a population health perspective and to identify ways to adequately manage them, such as using generally accepted methods of prioritizing contacts and the prevalence of infection in scaling up. Such involvement should result in further specification of the CI recommendations. Previous experience in guideline development and implementation has shown better acceptance when “owned and operated” by the profession itself [19]. In addition, enhancing PHNs to work from a population health perspective requires that the recommendations of the revised CI guidelines are explicit, build on pre-existing practices, and disseminated via comprehensive training that for instance included collaboration between PHNs and physician specialists in discussing and solving problems in guideline adherence [6]. Further research is needed to determine how revised guidelines can best be embedded in current practices.

Finally, our results indicate that the final outcome of a CI reported in the Netherlands Tuberculosis Register should be interpreted with caution. Nonadherence to the guidelines affected the CIs’ coverage and the yield. [20]. Coverage and yield data were predominantly obscured by PHNs by deliberately identifying more contacts and giving contacts a higher priority for testing than recommended, as well by misclassifying contacts, incorrectly excluding immunocompromised individuals for testing and incorrectly deciding about scaling up CIs. We observed incorrectly scaled up CIs, but in theory, CIs could also incorrectly not be scaled up due to a diluting effect of negative test results of unnecessarily tested contacts. Invalid coverage and yield data hamper monitoring and evaluation of policies at the national and European levels, where accuracy is essential for gaining more insight into transmission patterns and for appropriately revising current CI guidelines to further optimize TB control and public health expenditures [1].

In conclusion, we found limited adherence to the national CI guidelines for identifying and prioritizing contacts and scaling up a CI. For each step, several flaws and dilemmas were identified. Nonadherence was largely related to the ambiguity of the guidelines and to the tendency of the PHNs to act from an individual health-care position rather than a population health perspective. The usefulness of the stone-in-the-pond principle should be critically re-evaluated, especially among immigrant populations. Future development and dissemination of CI guidelines should appropriately address these difficulties an incorporate the continuous input of the TB workforce.
